# Human Motor Neurons With SOD1-G93A Mutation Generated From CRISPR/Cas9 Gene-Edited iPSCs Develop Pathological Features of Amyotrophic Lateral Sclerosis

**DOI:** 10.3389/fncel.2020.604171

**Published:** 2020-11-19

**Authors:** Byung Woo Kim, Jiwon Ryu, Ye Eun Jeong, Juhyun Kim, Lee J. Martin

**Affiliations:** ^1^Division of Neuropathology, Department of Pathology, Johns Hopkins University School of Medicine, Baltimore, MD, United States; ^2^Department of Neurology, Johns Hopkins University School of Medicine, Baltimore, MD, United States; ^3^Department of Psychiatry and Behavioral Sciences, Johns Hopkins University School of Medicine, Baltimore, MD, United States; ^4^The Solomon H Snyder Department of Neuroscience, Johns Hopkins University School of Medicine, Baltimore, MD, United States; ^5^Department of Anesthesiology and Critical Care Medicine, Johns Hopkins University School of Medicine, Baltimore, MD, United States

**Keywords:** ALS, SOD1, G93A, CRISPR/Cas9, iPSC, motor neuron, neurodegeneration

## Abstract

Amyotrophic lateral sclerosis (ALS) is a fatal neurodegenerative disorder characterized by gradual degeneration and elimination of motor neurons (MNs) in the motor cortex, brainstem, and spinal cord. Some familial forms of ALS are caused by genetic mutations in superoxide dismutase 1 (SOD1) but the mechanisms driving MN disease are unclear. Identifying the naturally occurring pathology and understanding how this mutant SOD1 can affect MNs in translationally meaningful ways in a valid and reliable human cell model remains to be established. Here, using CRISPR/Cas9 genome editing system and human induced pluripotent stem cells (iPSCs), we generated highly pure, iPSC-derived MNs with a SOD1-G93A missense mutation. With the wild-type cell line serving as an isogenic control and MNs from a patient-derived iPSC line with an SOD1-A4V mutation as a comparator, we identified pathological phenotypes relevant to ALS. The mutant MNs accumulated misfolded and aggregated forms of SOD1 in cell bodies and processes, including axons. They also developed distinctive axonal pathologies. Mutants had axonal swellings with shorter axon length and less numbers of branch points. Moreover, structural and molecular abnormalities in presynaptic and postsynaptic size and density were found in the mutants. Finally, functional studies with microelectrode array demonstrated that the individual mutant MNs exhibited decreased number of spikes and diminished network bursting, but increased burst duration. Taken together, we identified spontaneous disease phenotypes relevant to ALS in mutant SOD1 MNs from genome-edited and patient-derived iPSCs. Our findings demonstrate that SOD1 mutations in human MNs cause cell-autonomous proteinopathy, axonopathy, synaptic pathology, and aberrant neurotransmission.

## Introduction

Amyotrophic lateral sclerosis (ALS) is a progressive neurodegenerative disorder characterized by the gradual degeneration of motor neurons (MNs) leading to muscle weakness, atrophy, paralysis, and ultimately, respiratory failure and death (Le Verche and Przedborski, 2010). While mostly sporadic with unknown inheritance, about 10% of all ALS cases are familial. This molecular genetic pathology can provide important clues about the intrinsic and non-autonomous vulnerability of MNs (Boylan, 2015). Some familial ALS cases are linked to mutations in the superoxide dismutase 1 (SOD1) gene, encoding an antioxidant enzyme that functions as a homodimer, binding copper and zinc ions, to destroy superoxide radical (O_2_^–^) in the body (Petrov et al., 2016).

Despite being the first gene mutation identified in ALS (Rosen et al., 1993) and the extensive and important research on SOD1 and its putative disease mechanisms in ALS over the past decades, there are still no effective disease-modifying treatments for any form of ALS (Kawamata and Manfredi, 2010). Though the pathobiology of ALS is extraordinarily complex, historically research on disease mechanisms and experimental therapeutics has relied heavily on animal models that might not sufficiently replicate the human disease mechanisms and cellular neuropathology (Martin et al., 2007). Animal models, including SOD1-G93A transgenic mice, may not accurately model the genetics of ALS due to mutant gene copy numbers. Specifically, overexpression of mRNA or protein in some animal models and transfected cells make the models non-physiological, possibly producing phenotypes that are contrary to those observed from ALS patients with a single copy of a mutant *SOD1* gene (Ludolph et al., 2010; Philips and Rothstein, 2015; Morrice et al., 2018). Moreover, overexpression of wild-type human SOD1 causes axonopathy and mitochondrial vacuolation in mice (Jaarsma et al., 2000; Fischer et al., 2004).

To overcome this limitation, human induced pluripotent stem cells (iPSCs), promising sources of vulnerable and disease-relevant cell types, have enabled an opportunity to understand human disease modeling and mechanisms, and to explore human disease-relevant therapeutic development (Xu et al., 2013; Ohnuki and Takahashi, 2015). In addition, the evolution of genome editing systems allows creation of genetic modifications within cells with improved targeting efficiency and precision. Combination of these two approaches empowers investigation of human pathophysiology under the critically necessary human genomic background with all of the known and unknown genetic modifiers.

**FIGURE 1:**
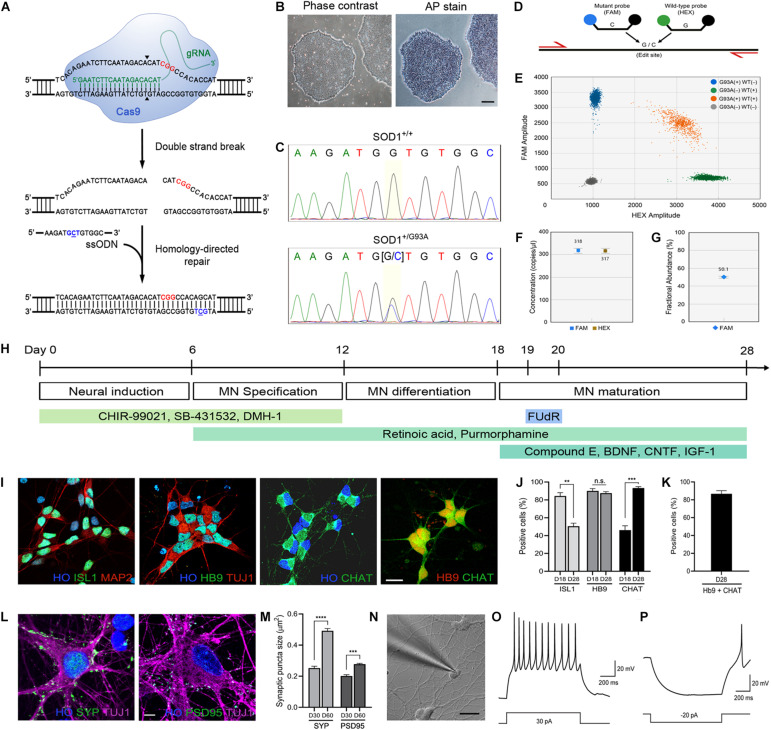
Generation of the *SOD1*^+/*G*93*A*^ iPSC line and differentiation into MNs. **(A)** Schematic representation of CRISPR/Cas9-mediated genome editing. The PAM sequence and the mutation site are shown in red and blue, respectively. **(B)** Phase-contrast image of human iPSCs and alkaline phosphatase staining showing stem cell pluripotency. Scale bar, 200 μm. **(C)** Sequencing chromatogram demonstrating CRISPR/Cas9-mediated genome editing of *SOD1^+/+^* to *SOD1*^+/*G*93*A*^. Homozygous nucleotide G in G93G and heterozygous nucleotides (G/C) in G93A are shown. **(D)** Schematic representation of homology-directed repair edit detection assay by ddPCR. A primer pair (red) amplifies the intended edit region. Mutant (FAM, blue) and wild-type (HEX, green) probes are designed to bind edited or unedited sequences, respectively. **(E)** Two-dimensional ddPCR scatter plot showing the four droplet clusters identified with a mutant and wild-type allele; FAM/HEX negative (gray), FAM positive (blue), HEX positive (green), and FAM/HEX positive (orange). **(F)** Concentrations of mutant and wild-type templates. **(G)** Quantification of SOD1-G93A mutation in the presence of wild-type DNA. CRISPR-induced G93A missense mutation was detected at frequency 50.2%. The error bars for **(F,G)** represents the Poisson 95% confidence intervals. **(H)** Schematic diagram showing the protocol used for MN directed differentiation from iPSCs. **(I)** Representative images of ISL1^+^, Hb9^+^, and ChAT^+^ MNs on day 28. Hoechst (HO) was used to counterstain the cell nuclei. Scale bar, 20 μm. **(J)** Quantification of ISL1^+^, Hb9^+^, and ChAT^+^ MNs on day 18–28. **(K)** Quantification of cells stained for both Hb9 and ChAT. **(L)** Representative images of SYP^+^ presynaptic and PSD95^+^ postsynaptic elements, respectively, on TUJ1^+^ MNs on day 60. Scale bar, 5 μm. **(M)** Quantification of SYP^+^ presynaptic and PSD95^+^ postsynaptic structures in MN cultures on day 30–60. All data for **(J,K,M)** are shown as means ± SEM. **(N)** Whole-cell patch clamp recording on a MN on day 28. Scale bar, 20 μm. **(O)** Representative voltage response showing repetitive action potentials to depolarizing current step. **(P)** Representative hyperpolarizing voltage response to negative current injection. **p < 0.01, ***p < 0.001, and **** *p* < 0.0001.

Here, we hypothesized that introduction of a disease-causing SOD1-G93A mutation to human iPSCs will phenocopy spontaneously occurring human ALS neuropathology after their differentiation into MNs. To test the hypothesis, we utilized CRISPR/Cas9 genome editing technology on iPSCs and introduced a SOD1-G93A missense mutation by performing knock-in into the genome (Figure 1A). By combining genome editing with stem cell differentiation approaches, we have generated highly pure MN lines that are “isogenic” to each other. With a patient-derived iPSC line harboring an A4V mutation as a positive control, we identified several disease phenotypes in these MNs relevant to ALS, including proteinopathy, axonopathy, synaptic pathology, and aberrant neurotransmission. Our findings demonstrate that genome edited iPSCs using CRISPR/Cas9-mediated targeted gene editing and their differentiation into MNs are important tools with their proper control cells to replicate and model ALS molecular, genetic, and neuronal pathology and to study mechanisms of disease in human ALS in cell culture.

## Materials and Methods

### iPSC Culture

The institutional biosafety committee (JHU registration B1011021110) approved the use of human cells. The protocols met all ethical and safety standards for work on human cells. All iPSC lines used in this study are listed in Supplementary Table S1 and were characterized in previous studies (Wen et al., 2014; Li et al., 2015; Kim et al., 2020). The human iPSC lines were maintained on culture plates coated with Matrigel (Corning) in StemFlex medium (Gibco) and passaged every 4–6 days by EDTA dissociation buffer (Beers et al., 2012).

### Mouse and Human Primary Cell Culture

Mouse cortical astrocytes were used to enhance attachment of iPSC-derived MNs for subsequent experiments including patch clamp recording. They were isolated from P3 to P4 CD1 mouse pups as described (Schildge et al., 2013) and cultured in Dulbecco’s modified Eagle’s medium (DMEM, Corning) supplemented with 10% FBS. Mouse embryonic fibroblasts (MEFs) were obtained from CF-1 mouse embryos at approximately 13.5 days gestation and cultured in DMEM with 10% fetal bovine serum (FBS, Hyclone), 1% GlutaMAX (Gibco), and 1% Minimum Essential Medium Non-Essential Amino Acids (MEM-NEAA, Gibco). MEFs were irradiated prior to culturing with iPSCs. Human primary astrocytes were purchased from Thermo Fisher Scientific. They were plated and cultured a week before MEA experiments in DMEM with 1% N2 supplement, and 10% FBS.

### Alkaline Phosphatase Staining

An alkaline phosphatase stain (Sigma) was used to identify undifferentiated, pluripotent iPSCs (Figure 1B). Briefly, iPSCs were washed twice with PBS and fixed in 4% paraformaldehyde solution for 10 min. The cells were washed again with PBS and incubated in SIGMA*FAST* BCIP/NBT (5-bromo-4-chloro-3-indolyl phosphate/nitro blue tetrazolium) solution (Sigma, 1 tablet in 10 mL) for 10–20 min at room temperature until the color disclosure. Solution was then removed and the cells were washed with PBS. Following the wash, cells were imaged by microscopy.

### CRISPR/Cas9 Genome Editing and Validation

Introduction of SOD1-G93A mutation using CRISPR/Cas9 (Figure 1A) was done using the indicated guide RNA and donor DNA as described by us previously (Kim et al., 2020). This cell line, along with its isogenic wild-type control and patient-derived iPSCs with SOD1-A4V mutation, were used in this study. Detection of wild-type and mutant alleles and copy number determination of genomic SOD1-G93A mutations in edited iPSCs were done using digital droplet PCR (ddPCR) (Figure 1D). Reaction mix was first prepared by adding isolated genomic DNA, 1X ddPCR supermix, 1X target (FAM-labeled) and wild-type (HEX-labeled) primers/probe (Supplementary Table S2), *Mse*I restriction enzyme, and H_2_O (Figure 1D). This was then loaded into DG8 cartridge along with droplet generation oil. The cartridge was placed in the QX200 droplet generator (Bio-Rad) for droplet generation. After droplets were generated, they were transferred into a 96-well plate and thermal cycling was used to amplify the sample. The plate containing the amplicons in droplets was subsequently placed and run in the QX200 Droplet Reader (Bio-Rad). Data analysis was done using the Quantasoft software (Bio-Rad) with at least 10,000 droplets.

The verification of isogenicity of the mutant and the wild-type iPSC lines after the genome editing was done using short tandem repeat (STR) profiling analysis. Thirteen STP loci (CSF1PO, FGA, TH01, TPOX, VWA, D3S1358, D5S818, D7S820, D8S1179, D13S317, D16S539, D18S51, D21S11) and amelogenin (AMEL) for sex determination were interrogated. Patient-derived iPSCs with an A4V mutation served as a control. Genomic DNA was extracted from all cell lines (Qiagen) according to the manufacturer’s instructions and the STR loci and the amelogenin locus were subsequently amplified using PCR primer pairs which were assessed previously (Azari et al., 2007). The 50 μL reaction mixture consisted of 100 ng genomic DNA, 1X standard *Taq* reaction buffer (NEB), 0.2 μM each primer, 200 μM dNTP, and 1.25 unit of Taq DNA polymerase (NEB). The PCR program started with an initial denaturation step at 95°C for 3 min, followed by 35 cycles of denaturation at 95°C for 30 s, annealing at 60°C for 30 s, and extension at 72°C for 45 s. The final extension step was at 72°C for 2 min. This protocol allowed simultaneous genotyping of all STRs and AMEL in a single PCR amplification. To acquire better resolution for separation of the PCR products, they were loaded onto a 3.5% NuSieve GTG Agarose gels (Lonza). The size of the gels was 14 cm x 12 cm x 1 cm. DNA electrophoresis was performed for 6 h at 110 V and visualized by ethidium bromide staining and ChemiDoc Imager (Bio-Rad).

For off-target analysis, top seven candidates were selected based on COSMID web tool (Cradick et al., 2014) The list of oligonucleotide sequences and the summary of off-target analysis were summarized in our previous study (Kim et al., 2020). The genomic DNA was isolated from iPSCs and PCR amplification was performed at loci of the seven sites. PCR amplicons were checked for any possible off-targets by direct DNA sequencing.

### Generation of Human MNs

Directed differentiation of MNs from human iPSCs (Figure 1H) was carried out as previously described (Kim et al., 2020). Briefly, iPSCs were passaged on irradiated MEF feeder layers in ESC medium (DMEM/F12, 20% KnockOut Serum Replacement (Gibco), 1% MEM-NEAA, 1% GlutaMAX, 10 ng/mL bFGF (PeproTech), 0.1 mM β-mercaptoethanol (Gibco), and 10 μm Y-27632 ROCK inhibitor) overnight. For neural patterning, the medium was replaced with modified N2/B27 medium (DMEM/F12:Neurobasal [1:1], 0.5% N2, 0.5% B27, 0.1 mM ascorbic acid, and 1% GlutaMAX) containing 3 μM CHIR-99021 (Tocris), 2 μM SB-431532 (Tocris), and 2 μM DMH-1 (Tocris) and cultured for 6–7 days. The neural progenitor cells (NPCs), which were grown in cell clusters, were then detached using 0.1% (w/v) collagenase IV (Gibco) and plated on Matrigel-coated plates in the media with 1 μM CHIR-99021, 2 μM SB-431532, 2 μM DMH-1, 0.1 μM retinoic acid (RA, Sigma), and 0.5 μM purmorphamine (Stemgent). After culturing the cells for additional 6–7 days for MN specification, MN progenitors (MNPs) were collected by collagenase IV and further differentiated in the modified N2/B27 medium supplemented with 0.5 μM RA and 0.1 μM purmorphamine (PUR). These steps specified the differentiating neuroprogenitors to MN identity (Wichterle et al., 2002) through caudalization (RA) and ventralization (PUR) as verified by specific markers (Supplementary Table S3). To differentiate MNs to more maturity, the cell clusters were dissociated into single cells with Accutase (Gibco) and plated either on Matrigel-coated plates or co-cultured with mouse primary astrocytes for 10 more days with 0.5 μM RA, 0.1 μM PUR, 0.1 μM Compound E (Millipore), 10 ng/ml brain-derived neurotrophic factor (BDNF, PeproTech), 10 ng/ml ciliary neurotrophic factor (CNTF, PeproTech), and 10 ng/ml insulin-like growth factor 1 (IGF-1, PeproTech). Non-motor neuron cell population, which includes undesired cell types and/or poorly differentiated cells, was prevented from further proliferation by addition of a mitotic inhibitor, 5-Fluoro-2′-deoxyuridine (FUdR).

### Alternative Protocol for MN Differentiation

Differentiation induction of cholinergic neurons from human iPSCs was carried out using the Quick-Neuron^TM^ Cholinergic—SeV Kit (Elixirgen Scientific, Inc., Baltimore, MD). The protocol was used because it is overall more rapid in generating differentiated MNs. This differentiation of human iPSCs into cholinergic neurons is driven by a Sendai virus (SeV)-based vector (ID Pharma Co., Ltd., Tokyo, Japan) which allows forced overexpression of neural inducing factors (Goparaju et al., 2017) in a temperature-dependent manner (Ban et al., 2011). Briefly, human iPSCs were infected with the SeV vector and incubated at 33°C, 5% CO_2_ for 2 days in differentiation medium for 2 days. Then, the cultures were transferred to 37°C, 5% CO_2_ to inactivate the SeV vector and further incubated for 16–18 h. Finally, immature neurons were passaged onto a coated microelectrode array plate, CytoView MEA (Axion Biosystems), after being mixed with human primary astrocytes (Thermo Fisher Scientific). This protocol has been shown to yield cholinergic MNs as identified by islet1 and Hb9 staining in combination with choline acetyltransferase (ChAT) staining (Goparaju et al., 2017).

### MEA Recording

Approximately 60,000–80,000 wild-type control or ALS mutant MNs along with 15,000–20,000 human primary astrocytes in 10 μL differentiation medium were plated on each well of a poly-l-ornithine-coated CytoView MEA 48-well plate. Glia-MN co-culture was used to allow for better uniform dispersal of MNs throughout the cultures for improved global recording within the wells. After 1–3 h of allowing the cells to settle, additional 300 μL of the medium was added to each well and further incubated at 37°C, 5% CO_2_. Neuronal activity was recorded using Maestro Pro MEA system and AxIS Navigator software (Axion Biosystems). To exclude the possibility of the MEA signals being artifacts, tetrodotoxin (TTX) was used as a negative control to block action potentials. Following baseline recording, vehicle (H_2_O) and two different concentrations of TTX (final concentrations 2.5 and 25 nM) were added sequentially to the wells and the activity was recorded for 600 s after each treatment. Additionally, recordings were made on wells with only astrocytes plated to determine if astrocytes are contributing to activities. All the collected data were digitized and analyzed with NeuralMetric Tool and AxIS Metric Plotting Tool (Axion Biosystems).

### Whole-Cell Patch Clamp Recording

Differentiated MN cultures on round coverslips (12 mm diameter) at days *in vitro* (DIV) 28 were placed in a submersion recording chamber on an upright microscope (Zeiss AxioExaminer Z1, Objectives: 5x, 0.16 NA and 40x, 1.0 NA) fitted for infrared differential interference contrast (IR-DIC) microscopy. The recording chamber was continuously superfused (2–4 ml/min) with artificial cerebrospinal fluid (aCSF) composed of (in mM): 125 NaCl, 26 NaHCO_3_, 2.5 KCl, 1.25 NaH_2_PO_4_, 1 MgSO_4_, 20 glucose, 2 CaCl_2_, 0.4 ascorbic acid, 2 pyruvic acid, and 4 L-(+)-lactic acid; pH 7.3, 315 mOsm, continuously bubbled with 95% O_2_/5% CO_2_. Neurons were visualized with a digital camera (Sensicam QE; Cooke). Glass recording electrodes (2–4 MΩ) were filled with an internal solution containing (in mM): 2.7 KCl, 120 KMeSO_4_, 9 HEPES, 0.18 EGTA, 4 MgATP, 0.3 NaGTP, 20 phosphocreatine (Na), pH 7.3, 295 mOsm. Cell electrical activities were obtained using a Multiclamp 700B amplifier (Molecular Devices) and an ITC-18 interface unit (Instrutech), both controlled using a customized routine written by Igor Pro (Wavemetrics). Action potentials were evoked by 1 s-long 10–100 pA current injections into cells. Hyperpolarizing responses were investigated by −20 pA current injections into cells. In a subset of current-step test, cell membranes were held at ∼80 mV to see action potential initiation more clearly. All signals were low-pass filtered at 10 kHz and sampled at 20 kHz.

### Extraction of Misfolded and Aggregated SOD1

Approximately 6 × 10^6^ cultured MNs for each line were used. Cells were first washed with PBS, collected by using a cell scraper, and harvested by centrifugation for 5 min at 200 g, 4°C. RIPA buffer with protease inhibitors was added to the pellet and the cells were incubated for 20 min on ice. During the incubation, probe sonication was performed briefly for 2–3 times to disrupt cell membranes and homogenize the samples. The lysates were centrifuged for 10 min at 14,000 g, 4°C and the supernatant (RIPA-soluble fraction) was collected. The pellet was washed with PBS and centrifuged again with the same condition. Urea buffer (8 M Urea, 4% CHAPS, 40 mM Tris, 0.2% BioLyte 3/10 ampholyte, 2 mM tributylphosphine) with protease inhibitors was then added to the pellet. After incubation for 30 min at room temperature, the samples were centrifuged for 10 min at 14,000 g, 4°C and supernatant (Urea-soluble fraction) was collected for SDS-PAGE and western blotting.

### MN Cell Body Size, Axon Length, and Process Branch Point Measurements

MNs spheroids, derived from treatment with small molecules (Figure 1H), on day 18 were individualized into single cells with Accutase, plated on coverslips in 24 well plates, and grown for additional 2, 4, and 6 days in cell culture. Cells were fixed for 10 min with 4% paraformaldehyde at each time point and immunostained with antibodies (Supplementary Table S3) to neuronal markers including TUJ1, MAP2, and Tau. Cell body sizes of MNs of each cell line at 4 days after plating were measured using ImageJ. For axon length measurement, an axon of a cell was first identified by a neurite that is Tau-positive but MAP2-negative. Using ImageJ, the length of the neurite was quantified. Only those neurons with axons that had more than twice the length of cell body were selected and measured. To count the number of branch points of both dendrites and axons, tracings of individual cells were first performed. This was done semi-automatically by using the NeuronJ plugin of ImageJ with fluorescence images taken 4 days after plating.

### Quantification of Synapse Size and Density

**FIGURE 2:**
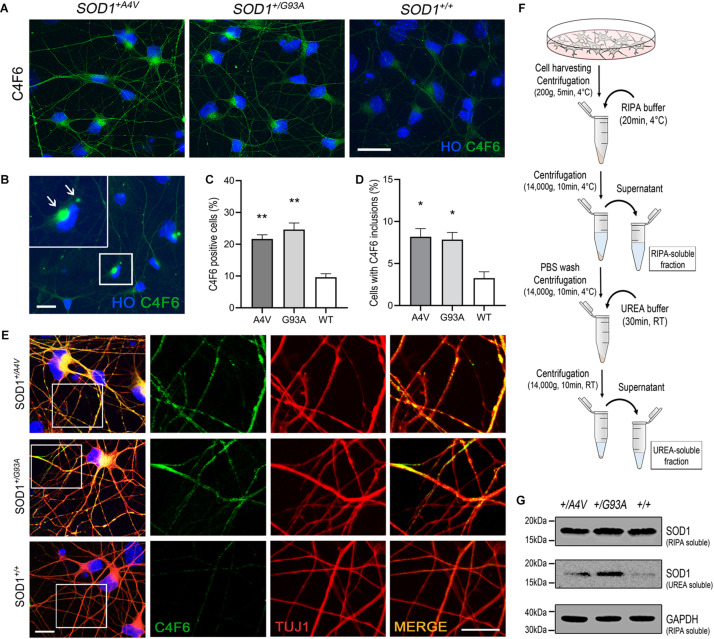
Detection of misfolded and aggregated SOD1 in MN cultures. **(A)** Immunofluorescence images of MNs showing C4F6 immunoreactivity, a marker for misfolded SOD1. Scale bar, 20 μm. **(B)** Immunofluorescence of revealed C4F6-positive inclusions and diffuse cytoplasmic immunoreactivity in mutant MNs. Scale bar, 20 μm. **(C,D)** Quantification of **(C)** C4F6-positive MNs with cytoplasmic immunoreactivity and **(D)** MNs with C4F6 inclusions. *n* = 3 biological replicates (a total of 949 cells with *SOD1*^+/*A*4*V*^ mutation, 876 cells with *SOD1*^+/*G*93*A*^ mutation, and 1072 cells with *SOD1^+/+^* were evaluated). All data shown as means ± SEM. *p < 0.05, **p < 0.01. **(E)** Immunofluorescence images of MNs showing the localization of misfolded SOD1 in processes of MNs visualized by TUJ1. Scale bars, 10 μm. **(F)** Schematic of experimental design for extracting RIPA-soluble and UREA-soluble fractions from MN cultures. **(G)** Western blots showing SOD1 in RIPA-soluble and UREA-soluble fractions. GAPDH was used as a loading control. *n* = 3 biological replicates.

MNs on day 60, a time at which synapses are well developed and prominent (Figure 2M), were fixed and immunostained with the anti-synapsin and anti-PSD95 antibodies (Supplementary Table S3). Fluorescence images of MNs were taken and analyzed using ImageJ. The area of each puncta was measured for quantifying synapse size. Images used for analyses were thresholded with a constant value for each channel. For synaptic density, the number of puncta per 100 μm was measured using a plugin, Puncta Analyzer.

### Measurement of Cellular ATP Level

Total cellular levels of ATP of iPSC-derived MNs were measured using ViaLight Plus kit (Lonza) according to manufacturer’s instruction. Dissociated 20,000 cells were plated in each well of Matrigel-coated 96-well plates. On the day of measurement, cells were first washed with PBS and lysed for 20 min in cell lysis reagent on a shaker to extract ATP. The cell lysates were then transferred to a 96-well clear bottom white polystyrene microplate (Corning) and ATP monitoring reagent plus was added to generate luminescent signal. After 1–2 min, the plate was placed in LMax II luminometer (Molecular Devices) and the ATP levels were measured with SoftMax Pro software (Molecular Devices).

### Immunofluorescence Staining

Cells were fixed in 4% paraformaldehyde for 10 min at room temperature and washed three times with PBS. Following fixation, cells were permeabilized with 0.2% Triton X-100 for 10 min, blocked in 10% donkey serum in PBS for 1 h, and incubated in primary antibodies overnight at 4°C. Cells were then washed in PBS, incubated with secondary antibodies, and stained with Hoechst 33258 DNA dye for nuclear visualization. See Supplementary Table S3 for a full list of antibodies.

### Western Blotting

Protein concentrations were first determined by Pierce BCA Protein Assay Kit (Thermo Fisher Scientific) and samples were loaded and separated by SDS-PAGE. For RIPA- and UREA-soluble fractions, 25 μg of proteins from RIPA-soluble fractions and equivalent volumes of proteins from UREA-soluble fractions were used. Proteins were transferred onto nitrocellulose membranes, washed with a blocking buffer, and probed with primary antibodies overnight at 4°C. The primary antibodies used are listed in Supplementary Table S3. The membrane was washed three times in the blocking buffer for 5 min and probed with secondary antibody conjugated with HRP (1:10,000, Invitrogen) for 1 h. Bands were detected with Pierce ECL western blotting substrate (Thermo Fisher Scientific).

### Microscopy and Image Acquisition

Cells on coverslips were mounted in ProLong Gold Antifade Mountant (Thermo Fisher Scientific, P36930) and confocal images were taken and analyzed using Leica TCS SP8 microscope and LAS X software (Leica, Germany). Fluorescence images were obtained by Keyence BZ-X700 fluorescence microscope. For phase-contrast images, Nikon Eclipse TS100 was used.

### Statistical Analysis

Statistical analyses were performed by using Student’s *t*-test and one-way analysis of variance using GraphPad Prism. All data are represented as mean ± SEM. Statistical significance was considered when *p*-value was less than 0.05 (^∗^*p* < 0.05, ^∗∗^*p* < 0.01, ^∗∗∗^*p* < 0.001, and ^****^p < 0.0001).

## Results

### Genome-Edited Human iPSCs, Patient-Derived iPSCs, and Isogenic Control iPSCs Are Efficiently Differentiated Into Highly Pure MNs

As G93A is the most widely studied SOD1 mutation in animal models including transgenic mice (Gurney et al., 1994; Pan et al., 2012) and A4V is one of the most common and aggressive SOD1 mutation in North America (Chen et al., 2014; Fay et al., 2016), we selected iPSC lines harboring these mutations for our study.

We introduced the SOD1-G93A missense mutation by CRISPR/Cas9 genome editing (Figure 1A) into a healthy control iPSC line (C3–1) (Wen et al., 2014). The pluripotency of iPSCs was verified by alkaline phosphatase staining prior to genome editing (Figure 1B). Genome editing was carried out by electroporation of cells with Cas9 nuclease and a guide RNA that specifically targets wild-type *SOD1* allele along with a single-stranded donor oligonucleotide (ssODN) (Figure 1A). After isolating and expanding single clones, a heterozygous SOD1-G93A mutation was validated in cells by Sanger sequencing (Figure 1C) and off-target analysis was done to make sure the mutation was introduced only at the locus of our interest (Kim et al., 2020). To further confirm the copy number of wild-type and mutant alleles, we performed droplet digital PCR (Figure 1D). As shown in Figures 1E–G, each allele was present as one single copy at a ratio near 1:1. The isogenicity of the two iPSC lines was verified by STR profiling analysis. The amelogenin locus for sex determination confirmed same sex. The 13 STR loci (CSF1PO, FGA, TH01, TPOX, VWA, D3S1358, D5S818, D7S820, D8S1179, D13S317, D16S539, D18S51, D21S11) were compared for patterns and sizes of PCR products of both cell lines with DNA gel electrophoresis. All 14 amplicons of one cell line matched with that of the other cell line, suggesting the unique identity of the two cell lines (Supplementary Figure S1). As a comparator to this mutant line, a patient-derived iPSC line with SOD1-A4V mutation (GO013) was used (Li et al., 2015). The SOD1-A4V line had PCR product patterns distinct from the other lines. Patient-derived iPSCs with SOD1-G93A mutation were unavailable.

Using these iPSC lines, we used directed differentiation with small molecule morphogens to generate human MNs (Kim et al., 2020). iPSCs were differentiated efficiently into MNs within 28–30 days (Figure 1H). The designation of MN was established by the multipolar morphology, relatively large cell body size and large open nucleus and by immunofluorescence staining using three different MN markers, including an early MN marker, ISL1, and mature MN markers, Hb9 and ChAT (Figure 1I). At 18–21 days of differentiation, more than 80% of the cell population was ISL1 and Hb9 positive (84.4 ± 3.7% and 90.1 ± 2.6% for ISL1 and Hb9 positive cells, respectively) and by day 28–31, we obtained 93.3 ± 1.6% ChAT-expressing MNs (Figure 1J). Because Hb9 also identifies subsets of spinal interneurons (Chang and Martin, 2011), we also co-labeled the cells with Hb9 and ChAT and 86.7 ± 3.5% of cells were positive for both markers (Figure 1K). The MN cultures also showed robust positivity for the presynaptic bouton marker synaptophysin (SYP) and the postsynaptic marker postsynaptic density protein 95 (PSD95) (Figure 1L). The average size of presynaptic puncta was generally larger than that of postsynaptic puncta, and both SYP^+^ presynaptic puncta and PSD95^+^ postsynaptic puncta became more prominent in sizes and numbers over time (Day 30: 0.252 ± 0.007 μm^2^ and 0.201 ± 0.011 μm^2^ for SYP^+^ and PSD95^+^ puncta sizes, respectively, Day 60: 0.492 ± 0.015 μm^2^ and 0.277 ± 0.007 μm^2^ for SYP^+^ and PSD95^+^ puncta sizes, respectively), indicating maturation of synapses in the MN culture (Figure 1M).

We performed whole-cell patch clamp recording on differentiated human MNs and confirmed that the cells were electrophysiologically functional neurons (Figure 1N). As shown in Figures 1O,P, the neurons fired repetitive action potentials in response to depolarizing current step injections, and displayed hyperpolarization followed by post-rebound action potential in response to negative current injections. These data demonstrate that the human iPSC-derived differentiated neurons are functional MNs as defined by morphology, immunophenotyping, and electrophysiology.

### Misfolded and Aggregated SOD1 Accumulate in *SOD1*^+/*G*93*A*^ and *SOD1*^+/*A*4*V*^ MNs Under Basal Culture Conditions

SOD1 with missense mutations associated with familial ALS is known to be more prone to misfolding and aggregation (Johnston et al., 2000; Shinder et al., 2001; Wang et al., 2002; Elam et al., 2003). Although controversial, misfolded SOD1 has also been studied and described as a pathological feature found in sporadic ALS (Kabashi et al., 2007; Rotunno and Bosco, 2013). Using the pair of isogenic iPSC lines along with a patient-derived iPSC line with SOD1-A4V mutation, we examined the spontaneous presence of misfolded and aggregated SOD1 in differentiated human MNs under basal culture conditions. Immunofluorescence showed that approximately 20–25% of MNs (21.7 ± 1.3% and 24.6 ± 2.1% for *SOD1*^+/*A*4*V*^ and *SOD1*^+/*G*93*A*^ MNs, respectively) with either SOD1-A4V or SOD1-G93A mutation were positive for a well-characterized misfolded SOD1 antibody (Pickles et al., 2016) that detects an exon 4 epitope, C4F6 (Figures 2A,C). We also tested another misfolded SOD1-specific antibody, B8H10, that detects an exon 3 epitope (Pickles et al., 2016), and a small portion of MNs with SOD1-G93A mutation were positive (Supplementary Figures S2A,B). In addition, some MNs had prominent inclusions (Figures 2B,D; 8.2 ± 1.0% and 7.8 ± 0.9% for *SOD1*^+/*A*4*V*^ and *SOD1*^+/*G*93*A*^ MNs, respectively). MNs with wild-type SOD1 also showed a low level of reactivity and barely perceptible inclusions (9.6 ± 1.1% and 3.3 ± 0.8%, respectively), representing basal endogenous misfolding of wild-type SOD1 (Medinas et al., 2018). Misfolded SOD1 was also in the processes of MNs including dendrites and axons (Figure 2E). Interestingly, misfolded SOD1 in mutant MNs was often localized to discrete domains of processes (Figure 2E). These morphological results were confirmed by immunoblotting assays on RIPA detergent soluble and insoluble fractions obtained from MN cultures (Figure 2F). Compared to the wild-type SOD1 MN culture, the amount of detergent insoluble SOD1 in the UREA soluble fractions of *SOD1*^+/*G*93*A*^ and *SOD1*^+/*A*4*V*^ MNs was higher (Figure 2G).

### Axonopathy and Somal Attrition Emerge in Mutant MNs

**FIGURE 3:**
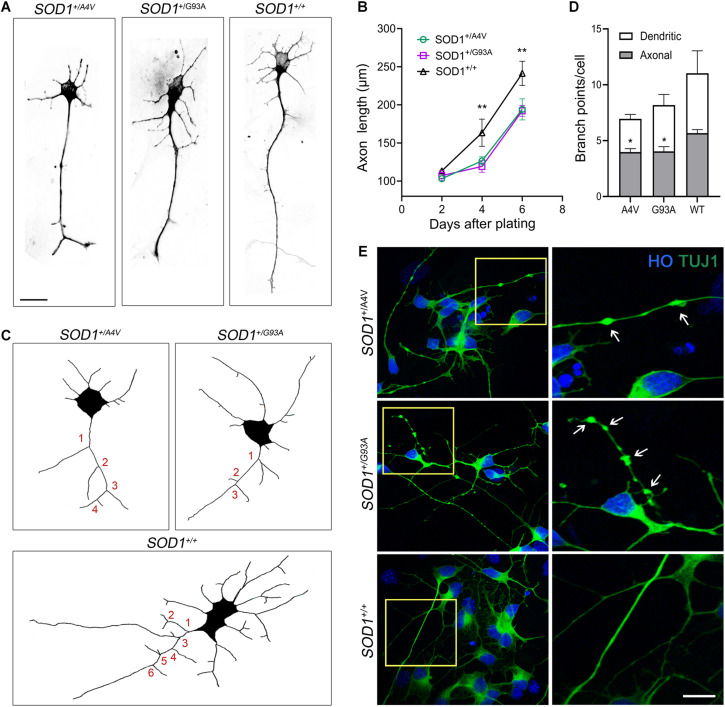
Axonal pathology and dystrophic neurites in *SOD1*^+/*A*4*V*^ and *SOD1*^+/*G*93*A*^ MNs. **(A)** Representative images of MNs showing different axonal lengths. Scale bar, 20 μm. **(B)** Quantification of average axonal length. *n* = 3 biological replicates for each time point (a total 556 cells with *SOD1*^+/*A*4*V*^ mutation, 429 cells with *SOD1*^+/*G*93*A*^ mutation, and 397 cells with *SOD1^+/+^* were evaluated). **(C)** Representative images of Tau^+^ axon tracing. Numbers in red indicate axonal branch points in each line. **(D)** Quantification of dendritic and axonal branch points on 6 day postplating. *n* = 4 biological replicates (a total of 258 cells with *SOD1*^+/*A*4*V*^ mutation, 186 cells with *SOD1*^+/*G*93*A*^ mutation, and 176 cells with *SOD1^+/+^* were evaluated). **(E)** Representative images of dystrophic neurites with swelling in the MN cultures on day 28. Arrows indicate swollen beads. Scale bar, 10 μm. All data shown as mean ± SEM. *p < 0.05, **p < 0.01.

As we observed misfolded and aggregated SOD1 in MN processes including axons, we assessed axonal pathology, which is a common feature of ALS (Carpenter, 1968; Delisle and Carpenter, 1984; Hirano et al., 1984; Fischer et al., 2004), in the mutant lines. We measured axonal length 2, 4, and 6 days after plating singlized MNs on day 18 and compared the mutants to the wild-type. At 2 days postplating, MNs did not manifest any difference in axonal length. However, the axonal length in the mutants was significantly shorter on 4 and 6 days postplating (Figures 3A,B; 4 days postplating: 126.3 ± 3.1 μm, 119.2 ± 4.4 μm, and 163.4 ± 10.3 μm for *SOD1*^+/*A*4*V*^, *SOD1*^+/*G*93*A*^, and *SOD1^+/+^* MNs, respectively, 6 days postplating: 194.1 ± 8.0 μm, 192.2 ± 4.1 μm, 241.3 ± 9.2 μm for *SOD1*^+/*A*4*V*^, *SOD1*^+/*G*93*A*^, and *SOD1^+/+^* MNs, respectively, *p* < 0.01, one-way ANOVA). Defects in axonal branching were also observed. MNs with SOD1-A4V and SOD1-G93A mutations had significantly fewer axonal branch points when compared to the wild-type (Figures 3C,D; 3.9 ± 0.3, 4.0 ± 0.4, and 5.7 ± 0.3 for *SOD1*^+/*A*4*V*^, *SOD1*^+/*G*93*A*^, and *SOD1^+/+^* MNs, respectively, *p* < 0.05, one-way ANOVA). Moreover, SOD1 mutant MNs formed neuritic swellings seen as focal enlargement of processes with a beaded or fragmented morphology (Figure 3E). In addition to axonal pathology, cell body attrition was observed. MNs with the mutations showed 20–25% reduction in soma size when compared to the wild-type (Supplementary Figures S3A,B; 140.6 ± 2.3 μm^2^, 140.9 ± 1.7 μm^2^, and 178.5 ± 3.2 μm^2^ for *SOD1*^+/*A*4*V*^, *SOD1*^+/*G*93*A*^, and *SOD1^+/+^* MNs, respectively, *p* < 0.01, one-way ANOVA).

### Mutant MNs Exhibit Postsynaptic Puncta Size Abnormalities and Synaptic Excess

**FIGURE 4:**
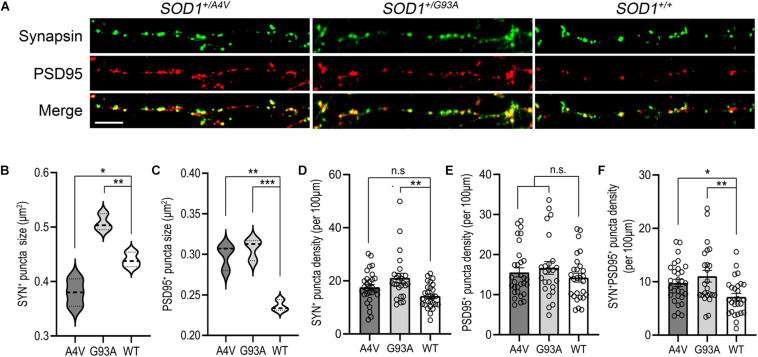
Synaptic structure abnormalities in *SOD1*^+/*A*4*V*^ and *SOD1*^+/*G*93*A*^ MNs. **(A)** SYN^+^ and PSD95^+^ immunofluorescence staining decorating MN processes with pre- and postsynaptic puncta. Scale bar, 10 μm. **(B,C)** Quantification of **(B)** SYN^+^ and **(C)** PSD95^+^ puncta sizes. **(D–F)** Quantification of **(D)** SYN^+^, **(E)** PSD95^+^, and **(F)** SYN^+^PSD95^+^ puncta density (per 100 μm). Each dot indicates a dendrite analyzed. All data shown as mean ± SEM. *p < 0.05, **p < 0.01, ***p < 0.001.

Synaptic dysregulation has been studied as an early pathological event contributing to motor deficits in ALS (Radzicki et al., 2016; Fogarty, 2019). We looked for synaptopathy in the mutant iPSC-derived MNs using immunofluorescence for presynaptic and postsynaptic markers (Figure 4A). The average synapsin-positive puncta size of *SOD1*^+/*A*4*V*^ MNs was smaller than the wild-type whereas that of *SOD1*^+/*G*93*A*^ MNs was larger (Figure 4B; 0.380 ± 0.015 μm^2^, 0.508 ± 0.009 μm^2^, and 0.440 ± 0.008 μm^2^ for *SOD1*^+/*A*4*V*^, *SOD1*^+/*G*93*A*^, and *SOD1^+/+^* MNs, respectively, *p* < 0.05, one-way ANOVA). For the size of postsynaptic puncta labeled by PSD95, however, both *SOD1*^+/*A*4*V*^ and *SOD1*^+/*G*93*A*^ MNs showed significantly larger puncta size than the wild-type (Figure 4C; 0.298 ± 0.009 μm^2^, 0.307 ± 0.008 μm^2^, and 0.236 ± 0.004 μm^2^ for *SOD1*^+/*A*4*V*^, *SOD1*^+/*G*93*A*^, and *SOD1^+/+^* MNs, respectively, *p* < 0.05, one-way ANOVA). The synaptic puncta density (number of puncta per 100 μm of dendritic length), was also quantified. There was a trend for pre- and postsynaptic puncta densities being relatively higher in mutant MN cultures compared to wild-type (Figures 4D,E), though not achieving significance (SYN^+^ puncta density: 17.8 ± 0.5, 21.2 ± 1.6, and 14.5 ± 1.1 for *SOD1*^+/*A*4*V*^, *SOD1*^+/*G*93*A*^, and *SOD1^+/+^*, respectively, PSD95 + puncta density: 15.6 ± 0.4, 16.8 ± 1.4, and 14.2 ± 1.1 for *SOD1*^+/*A*4*V*^, *SOD1*^+/*G*93*A*^, and *SOD1^+/+^*, respectively, *p* > 0.05, one-way ANOVA). We also carefully examined the density of synapses, specifically quantifying sites at which both synapsin and PSD95 colocalized. The numbers of SYN^+^PSD95^+^ puncta per 100 μm of dendritic surface were significantly greater in the mutants compared to wild-type MNs (Figure 4F; 9.8 ± 0.7, 11.0 ± 0.2, and 7.2 ± 0.2 for *SOD1*^+/*A*4*V*^, *SOD1*^+/*G*93*A*^, and *SOD1^+/+^* MNs, respectively, *p* < 0.05, one-way ANOVA).

### Electrophysiological Profiling of Mutant MNs Identifies Impairments in Functional Networks

**FIGURE 5:**
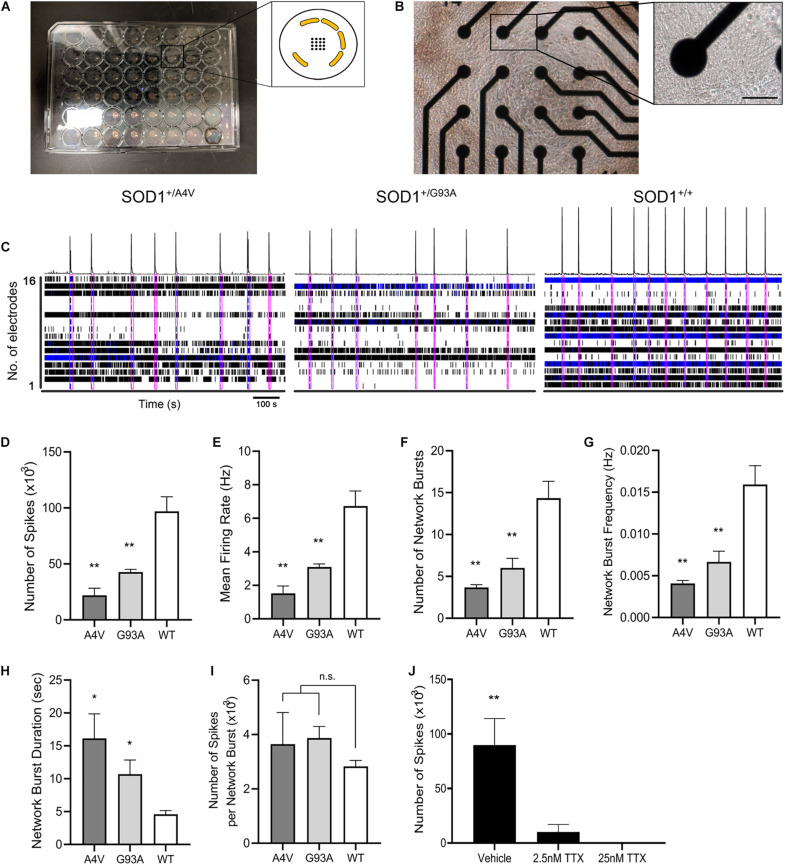
Functional networks are defective in *SOD1*^+/*A*4*V*^ and *SOD1*^+/*G*93*A*^ MN cultures. **(A)** Microelectrode array plate with 16 electrodes in the center of each well. Electrodes (black) are arranged in a 4 × 4 grid with peripheral grounds (yellow). **(B)** Phase-contrast image of iPSC-derived MNs cultured on the plate in each well. Scale bar, 50 μm. Enlarged image shows a single electrode in contact with MNs in culture. **(C)** Spontaneous firing of action potentials shown as raster plots and spike histogram during 900 s episodes. Pink boxes indicates network bursts. **(D)** Total number of spikes, **(E)** mean action potential firing rates (Hz), **(F)** total number of network bursts, **(G)** network burst frequency (Hz), **(H)** average network burst duration (s), and **(I)** average number of spikes in a network burst during 900 s episodes. **(J)** Effects of TTX treatment on neuronal activity. MEAs were done on MNs at 72 DIV for **(A–I)** and 226 DIV for **(J)**. *n* = 3 biological replicates. All data shown as mean ± SEM. *p < 0.05, **p < 0.01.

Because individual mutant MNs showed defects in axons and synapses, we tested whether electrophysiological functional network activity was correspondingly aberrant. We used microelectrode arrays (MEA) that allow repeated and simultaneous extracellular recordings of cells in a non-invasive manner (Hofmann and Bading, 2006; Massobrio et al., 2015; Odawara et al., 2016). MNs differentiated using the alternate protocol were characterized for cell type and enrichment by immunofluorescence staining of MAP2, Hb9, and ChAT, and their quantification is shown (Supplementary Figures S4A,B). Wild-type control or ALS mutant MNs along with human primary astrocytes were plated and cultured in MEA 48-well plate, covering the electrodes in each well (Figures 5A,B). Comparison of the total number of actions potentials and spike frequencies showed significantly less spontaneous firing of action potentials (21935.3 ± 6370.3, 42708 ± 2507.9, and 96907.3 ± 12988.3 for *SOD1*^+/*A*4*V*^, *SOD1*^+/*G*93*A*^, and *SOD1^+/+^* MNs, respectively, *p* < 0.01, one-way ANOVA) with lower mean firing rates (Figures 5C–E; 1.5 ± 0.4 Hz, 3.1 ± 0.2 Hz, and 6.7 ± 0.9 Hz for *SOD1*^+/*A*4*V*^, *SOD1*^+/*G*93*A*^, and *SOD1^+/+^* MNs, respectively, *p* < 0.01, one-way ANOVA) in the mutants during the recording episode. Network bursting of these MNs was also interrogated. Network bursting, an intermittent and synchronized network-wide bursts of action potentials characterized by brief periods of intense and multiple spikes (Fardet et al., 2018), is especially important as it provides a more comprehensive profile of electrophysiological activities of MN pools. The total number of network bursts was significantly smaller in the mutants (3.7 ± 0.3, 6.0 ± 1.2, and 14.3 ± 2.0 for *SOD1*^+/*A*4*V*^, *SOD1*^+/*G*93*A*^, and *SOD1^+/+^* MNs, respectively, *p* < 0.01, one-way ANOVA) and their bursting did not occur as frequently as the wild-type control (0.0041 ± 0.0003 Hz,

0.0067 ± 0.0013 Hz, and 0.0159 ± 0.0023 Hz for *SOD1*^+/*A*4*V*^, *SOD1*^+/*G*93*A*^, and *SOD1^+/+^* MNs, respectively, *p* < 0.01, one-way ANOVA), which corresponded to the less number of spikes and lower mean firing rate detected from individual MN pools (Figures 5C,F,G). These results suggest that the mutant MNs have impaired ability of firing action potential and reduced synchronized network firing. Interestingly, however, once network bursting is engaged, mutant MNs showed remarkably longer duration of bursting (Figure 5H; 16.1 ± 3.7 s, 10.7 ± 2.1 s, and 4.6 ± 0.6 s for *SOD1*^+/*A*4*V*^, *SOD1*^+/*G*93*A*^, and *SOD1^+/+^* MNs, respectively, *p* < 0.05, one-way ANOVA). Although not reaching significance, the average number of spikes per network burst in the mutants was also higher than the wild-type (Figure 5I; 3646.3 ± 1165.1, 3874.2 ± 423.8, and 2825.0 ± 223.8 for *SOD1*^+/*A*4*V*^, *SOD1*^+/*G*93*A*^, and *SOD1^+/+^* MNs, respectively). To confirm that the signals detected are not due to artifacts, we treated the cells with TTX and recorded the plate. After the baseline recording, cells were treated with vehicle (H_2_O) and then with two different concentrations of TTX (final concentrations 2.5 and 25 nM) sequentially. The activities of MNs diminished drastically at 2.5 nM TTX treatment and with 25 nM TTX, no activities were detected, suggesting that the signals are solely from the neuron Na^+^ channel-driven action potentials (Figure 5J). In addition, in order to exclude the possibility that the astrocytes are contributing to the network activities, we recorded wells plated with only astrocytes and did not see any signals (data not shown).

## Discussion

We tested the hypothesis that human iPSCs expressing a heterozygous SOD1-G93A mutation generated by CRISPR/Cas9 genome editing and their differentiation into MNs could be used as a faithful cell system that develops autonomous ALS-relevant pathological phenotypes. The evidence indicates that this hypothesis was correct. The human genome-edited SOD1-G93A MNs, as well as MNs generated from a patient-derived iPSC line with SOD1-A4V, developed robust disease phenotypes including proteinopathy, structural attrition, axonopathy, synaptic pathology, and functional defects.

The human iPSCs harboring a SOD1-G93A missense mutation were generated by us using CRISPR/Cas9 for the first time (Kim et al., 2020). The cell line expresses a single allele of mutant SOD1-G93A, thus at physiological disease-relevant levels, with the corresponding human genetic background. This work is significant because we showed that the MNs derived from the cell line develop ALS-related disease features. The detailed phenotypic characterization demonstrated in this study is original and this novel cell line supports the needs for studying ALS mechanisms in a human cell system. It is especially important as the SOD1-G93A variant is one of the most common mutations in ALS and known to have relatively rapid disease progression (Kato, 2008). In addition, this human cell line can be a good comparison to the SOD1-G93A transgenic mouse (Gurney et al., 1994) which is the most widely used and characterized rodent model of ALS. However, despite the decades-spanning use of rodents in ALS research and preclinical therapeutics, the translation of this work on transgenic mice to clinical practices has been ineffective. A possible explanation for this failure is the high copy numbers of human SOD1-G93A expressed in transgenic animals. The SOD1-G93A mice are known for their severe mitochondrial damage including structural and biochemical pathology within MNs *in vivo* (Bendotti et al., 2001; Sasaki et al., 2004; Chang and Martin, 2009) and in primary cell culture (Chang and Martin, 2016). However, we did not observe obvious changes in mitochondrial morphology or protein levels for VDAC1 or SOD2 in human SOD1-A4V or SOD1-G93A MNs. Moreover, ATP production was enhanced significantly in the human mutant MNs compared to wild-type MNs throughout their maturation (Supplementary Figure S5). From this aspect, our CRISPR/Cas9 engineered iPSC-derived human MNs are a new and alternative tool that could generate novel information on ALS pathogenesis that is distinct from data gleaned from SOD1 mice.

As with primary neural cell cultures (Lesuisse and Martin, 2002), modeling human diseases with iPSCs has several technical challenges. Heterogeneous cells in culture including unwanted mixed neuronal cell types and poorly differentiated immature cells, and partially dying cells all contribute to variability. With cells of different origin, there are genetic differences between individuals (Chen et al., 2014). In ALS-related cell culture studies, for example, having MNs and other neuronal cell types as well as glia bearing the same ALS gene mutation confounds interrogation of MN autonomous pathophysiology. We employed directed differentiation of highly pure MNs using the principles of embryogenesis and small molecule morphogens to mitigate confounders of cell heterogeneity, thusly, identifying disease phenotypes specifically in human MNs. However, for morphological and electrophysiological studies it is often necessary to grow the MNs on feeder layers of normal astrocytes for optimal cultures. In this case, we used wild-type mouse astrocytes so these cells would not be confused with human MNs morphologically, and for MEA we used wild-type human astrocytes, and they did not contribute the MEA activity, shown to be dose-dependently blocked by TTX. However, the inherent limitation here is the reductionism to a single human cell type in culture devoid of the complexity of the human CNS with a heterozygous gene mutation being expressed in a variety of cell types. The re-establishment and renewal of MN autonomous concepts for degeneration using human MNs derived from iPSC lines could provide significant new information regarding relevant cell-type therapeutics for ALS. Our observations suggesting MN autonomous mechanisms of degeneration in human ALS SOD1 mutants are consistent with previous studies on transgenic mice showing that neuron-specific expression of human mutant SOD1 is sufficient to induce MN degeneration in mice (Jaarsma et al., 2008; Wang et al., 2008), while astrocyte-specific expression of mutant SOD1 did not cause disease (Gong et al., 2000). However, mouse cell culture experiments have shown that mutant SOD1 in astrocytes can precipitate degenerative changes in wild-type MNs and worsen degenerative in MNs harboring mutant SOD1 (Nagai et al., 2007). Additional cell culture experiments are needed using our human SOD1-A4V or SOD1-G93A MNs in co-culture with astrocytes to further explore if non-neuronal cells aggravate the pathology in MNs.

A prominent phenotype discovered in our mutant MNs was misfolded and aggregated SOD1. It was seen diffusely in the cell body and processes, including axons, and as discrete cytoplasmic inclusions (Figure 2). This morphological visualization of misfolded SOD1 aggregates in processes and distinct inclusions within MN cell bodies under basal condition has not been shown before in iPSC-derived MNs. These morphological findings were supported by biochemical corroboration with detection of SOD1 in the urea-soluble fraction. SOD1 aggregation and inclusion formation are salient pathologies seen in human ALS (Forsberg et al., 2019). Our finding that misfolded and aggregated SOD1 is present spontaneously in iPSC-derived MNs is novel because previously insoluble SOD1 protein was found only when the proteasome was inhibited artificially in iPSC-derived MNs (Kiskinis et al., 2014). This supports the idea that accumulation of misfolded and aggregated SOD1 is a spontaneous pathology occurring in the presence of endogenously functioning proteasome. Thus, a possible driver of ALS could be intrinsic to proteasome dysfunction. These results hint that therapeutic targeting of the proteasome with modulating agonists could be relevant to ALS. This idea can be tested in our new human iPSC cell line.

Because we observed misfolded and aggregated SOD1 in MN processes including axons, we examined our cell cultures for axonal pathology, which is a dominant feature of ALS neuropathology (Carpenter, 1968; Delisle and Carpenter, 1984; Hirano et al., 1984; Fischer et al., 2004). We found that *SOD1*^+/*A*4*V*^ and *SOD1*^+/*G*93*A*^ MNs develop prominent axonopathies identified as axonal length truncation, smaller number of branch points, and axonal swelling in the mutants when compared to the wild-type MNs (Figure 3). These findings are original and potentially significant. Mutant MNs with misfolded and aggregated SOD1 and axonopathy identified here are meaningful because of their possible relationship to perturbations in intracellular trafficking, but pursuit of such requires live-cell imaging. Previous studies have suggested that misfolded SOD1 disrupts ER-to-Golgi trafficking driven by COPII vesicles (Burk and Pasterkamp, 2019). Also, it has been shown that mutant SOD1 inhibits kinesin- and dynein-mediated axonal transport (Huai and Zhang, 2019). As axonal transport is an essential cellular process responsible for the movement of molecular cargos including lipids, mitochondria, proteins, and cellular organelles in neurons (Burk and Pasterkamp, 2019), its defect has been implicated in ALS pathogenesis. Impaired transport increases stalling of cellular cargos along the axon, resulting in axonal swelling (Fassier et al., 2013). Axonopathy is also critical to MNs because they have long axons that innervate skeletal muscle fibers; sciatic nerve motor axons can be up to 1 meter in length. The aberrant axonal branching may disadvantage neuronal network formation and signal transmission (Suzuki et al., 2020). Shorter axonal length and diminished branching in the mutants could affect recruitment of myofibers during muscle contractions leading to weakness and fatigue. Neuromuscular junction (NMJ) number and integrity could also be compromised by these axonal perturbations (Kalil and Dent, 2014). As dismantling of NMJs perhaps plays a critical role in the onset of ALS (Lanuza et al., 2019), once reliable and quantitatively robust new human MNs cell co-culture systems with skeletal muscle cells are fashioned they could be important for exploring the dismantling of the NMJ and identifying therapeutics for rescuing distal axon disease phenotypes.

Dysregulation of synapses is thought to play a role in ALS (Wang et al., 2009; Jiang et al., 2019). By interrogating the synaptic compartment, we found, with time in culture and achievement of stable mature MNs, larger postsynaptic puncta sizes and higher synaptic puncta density in the mutants. These synaptic phenotypes, regarding synapse size and density, are novel discoveries for human iPSC-derived MNs. Synaptic phenotypes in human MN cell culture are relevant because they appear to be associated with the ALS disease progression (Sasaki and Maruyama, 1994; Shiihashi et al., 2017; Starr and Sattler, 2018). Consequently, in addition to axonopathy *per se*, the synapse has been receiving great attention as a promising therapeutic target for the disease (Murray et al., 2010; van Zundert et al., 2012; Casas et al., 2016; Cantor et al., 2018).

The larger postsynaptic puncta size was demonstrated by PSD95 which is a major member of the membrane-associated guanylate kinase family and known for regulating glutamate receptors at the synapse. The size and intensity of PSD95 puncta correlates with synaptic development and maturity of glutamatergic excitatory synapses (El-Husseini et al., 2000). In iPSC-derived MNs from C9ORF72 ALS/FTD patients, cell surface levels of NMDA receptor NR1 and the AMPA receptor GluR1 were increased and these receptors were accumulated at postsynaptic densities (Shi et al., 2018). Overexpression of TDP-43^A315*T*^ in mouse cortical neurons also showed elevated levels of GluR1 (Jiang et al., 2019). We also found that SYN^+^PSD95^+^synaptic puncta density was higher in the mutants. This finding mirrors results in which glutamatergic excitatory synaptic inputs and dendritic spine densities were increased in presymptomatic TDP-43^Q331*K*^ mice, consistent with the concept of excitotoxicity mechanisms in ALS (Fogarty et al., 2016). Our results on synaptic dysregulation in combination with other systems of ALS suggest that mutant SOD1 MNs exhibit advanced maturation of synapses compared to wild-type MNs. This abnormal development of synapses might be linked to a sentinel earlier cellular and molecular mechanism of ALS.

Lastly, our MEA results could be related to defects that we observed in the axons and synapses of the mutant MNs. Network bursting of the mutant MNs has possible relevance in generation of rhythmic motor patterns (Obien et al., 2014). Central pattern generators (CPGs), neural networks that drive rhythmic movements such as walking, chewing, and breathing without sensory feedback, have been correlated with ALS and dysfunction of CPGs has been found in ALS patients (Aydogdu et al., 2011). Mutant MNs having less number of network bursting with lower network burst frequency implies that their circuit formation locally or with other cell types in the central nervous system is abnormal, possibly due to pathologies or dysregulations that we found in the axons and synapses. Also, of note is that mutants showed longer duration with more numbers of spikes in a network burst.

ALS has aging as a key risk factor, with the symptoms usually developing between the ages of 55–70 (Martin et al., 2017). In Parkinson’s disease, similarly with aging as a strong risk factor, patients administered levodopa showed reduced burst duration as well as shorter amplitudes on electromyography (EMG), which correlated with the motor improvement (Tinkhauser et al., 2017). Similar results were shown by quantifying age-related differences in burst activity measured by EMG. Interestingly, muscles of old adults (adults > 70 years old) exhibited longer burst duration with higher mean amplitudes over an 8 h recording when compared to young adults (adults < 26 years old) (Theou et al., 2013). With this precedent in mind, our results on ALS MNs suggest that perturbations in MNs bursting patterns in cell culture could provide early prodromal mechanistic insight.

## Conclusion

In conclusion, our findings demonstrate that genome edited iPSCs using CRISPR/Cas9-mediated targeted gene editing and their differentiation into MNs provide important cellular tools to study mechanisms of disease in human ALS, including proteinopathy, axonopathy, synaptic pathology, and electrophysiological defects. This work can provide needed new insight into human cell-relevant therapeutic targets.

## Data Availability Statement

The raw data supporting the conclusions of this article will be made available by the authors, without undue reservation.

## Author Contributions

BK and LM conceived and designed the experiments, analyzed the data, and wrote the manuscript. JR assisted in stem cell maintenance and motor neuron differentiation. YJ took fluorescence images and performed ATP assay. JK conducted whole-cell patch clamp recordings and analyzed the data. All authors approved the final manuscript.

## Conflict of Interest

The authors declare that the research was conducted in the absence of any commercial or financial relationships that could be construed as a potential conflict of interest.

## Supplementary Material

Supplementary Figure 1 Gel electrophoresis of 13 STR loci and amelogenin. The separation of PCR products on 3.5% Nusieve GTG agarose gels. Total of 13 STR loci (CSF1PO, FGA, TH01, TPOX, VWA, D3S1358, D5S818, D7S820, D8S1179, D13S317, D16S539, D18S51, D21S11) and amelogenin for sex determination were used for STR profiling analysis. M: DNA marker, A: PCR products from cells with SOD1-A4V mutation, B: PCR products from cells with SOD1-G93A mutation, C: PCR products from the wild-type cell line.

Supplementary Figure 2 SOD1 misfolding and aggregation detected by B8H10 antibody in MN cultures. **(A)** Immunofluorescence images of misfolded SOD1 detected by B8H10 monoclonal antibody in MN culture. Scale bar, 20 μm. **(B)** Quantification of B8H10-positive cells. *n* = 3 biological replicates (a total of 386 cells with *SOD1*^+/A4V^ mutation, 466 cells with *SOD1*^+/G93A^ mutation, and 401 cells with *SOD1*^+/+^ were evaluated). Data shown as mean ± SEM.

Supplementary Figure 3 Cell body attrition in *SOD1*^+/A4V^ and *SOD1*^+/G93A^ MNs. **(A)** Immunofluorescence images of TUJ1^+^ MNs with Hoechst (HO) DNA nuclear staining showing different soma sizes with contoured perimeters in white. Scale bar, 10 μm. **(B)** Quantification of soma size on day 28. *n* = 4 biological replicates (a total of 315 cells with *SOD1*^+/A4V^ mutation, 206 cells with *SOD1*^+/G93A^ mutation, and 266 cells with *SOD1*^+/+^ were evaluated). All data shown as mean ± SEM. ^∗∗^*p* < 0.01.

Supplementary Figure 4 Characterization of MNs for MEA. **(A)** Immuno- fluorescence images of MNs stained for MAP2, Hb9, and ChAT. Hoechst (HO) was used to counterstain the cell nuclei. Scale bar, 20 μm. **(B)** Quantification of MNs that are Hb9^+^, ChAT^+^, or both. All data shown as mean ± SEM.

Supplementary Figure 5 Mitochondrial localization and ATP level in *SOD1*^+/A4V^ and *SOD1*^+/G93A^ MNs. **(A)** Immunofluorescence images of hMito^+^ MNs showing distribution and morphology of mitochondria. Scale bar, 10 μm. **(B)** Western blots showing the levels of VDAC1 and SOD2, mitochondrial markers, in whole cell lysates of MNs. GAPDH was used as a loading control. **(C)** Normalized ATP levels of MNs measured for 12 weeks after motor neurons became Hb9 positive. *n* = 8 biological replicates. All data shown as mean ± SEM. ^∗^*p* < 0.05, ^∗∗^*p* < 0.01, ^∗∗∗^*p* < 0.001.

Supplementary Table 1 Human iPSC lines used to generate MNs.

Supplementary Table 2 Target and wild-type primers/probe list.

Supplementary Table 3 Primary antibody list.
