# Supplementary figures and images for: Human Motor Neurons With SOD1-G93A Mutation Generated From CRISPR/Cas9 Gene-Edited iPSCs Develop Pathological Features of Amyotrophic Lateral Sclerosis
Source: Front Cell Neurosci. 2020 Nov 19;14:604171. doi: 10.3389/fncel.2020.604171 (PMC7710664; doi:10.3389/fncel.2020.604171)

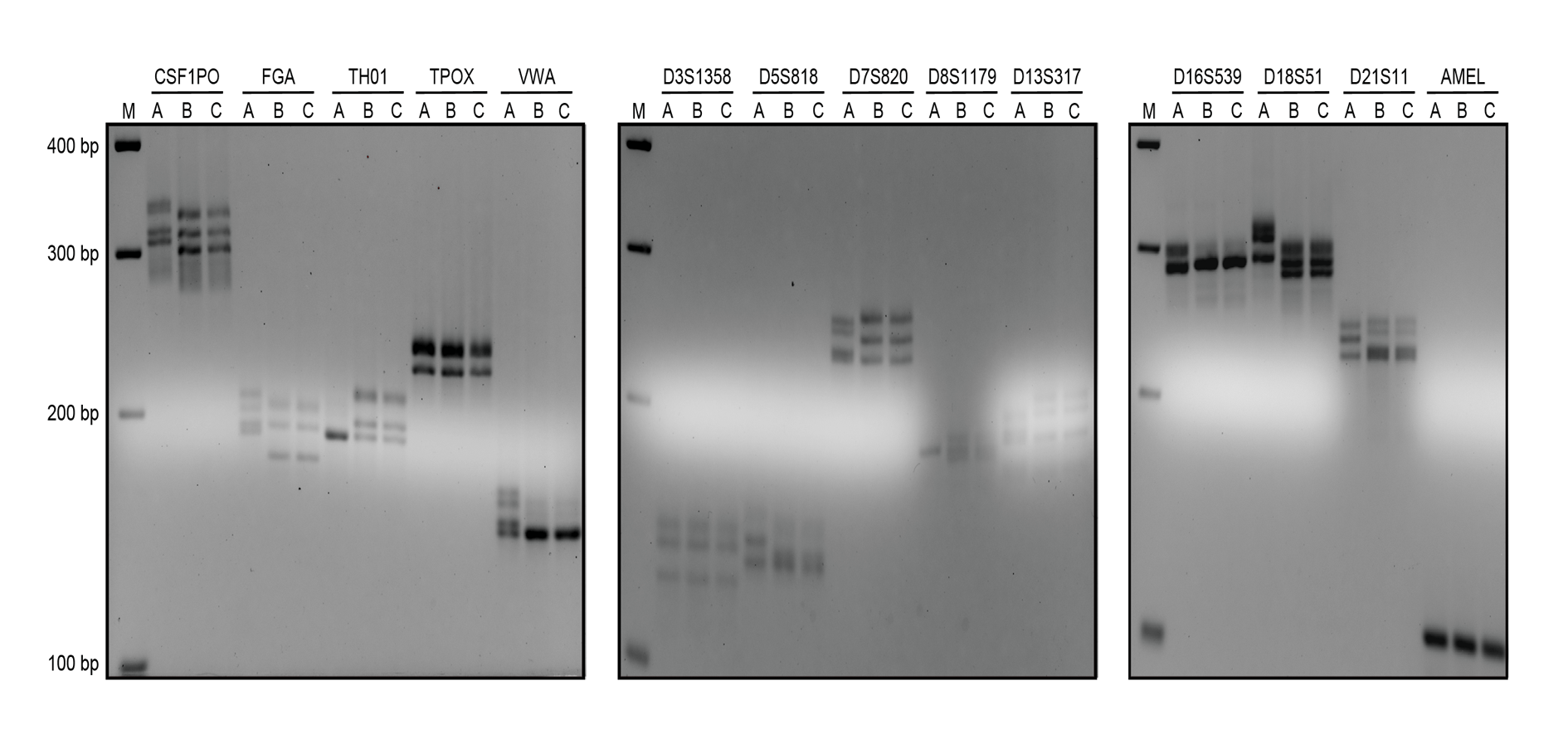

Supplement: Supplementary Figure 1 — Gel electrophoresis of 13 STR loci and amelogenin. The separation of PCR products on 3.5% Nusieve GTG agarose gels. Total of 13 STR loci (CSF1PO, FGA, TH01, TPOX, VWA, D3S1358, D5S818, D7S820, D8S1179, D13S317, D16S539, D18S51, D21S11) and amelogenin for sex determination were used for STR profiling analysis. M: DNA marker, A: PCR products from cells with SOD1-A4V mutation, B: PCR products from cells with SOD1-G93A mutation, C: PCR products from the wild-type cell line. [file Image_1.TIF]

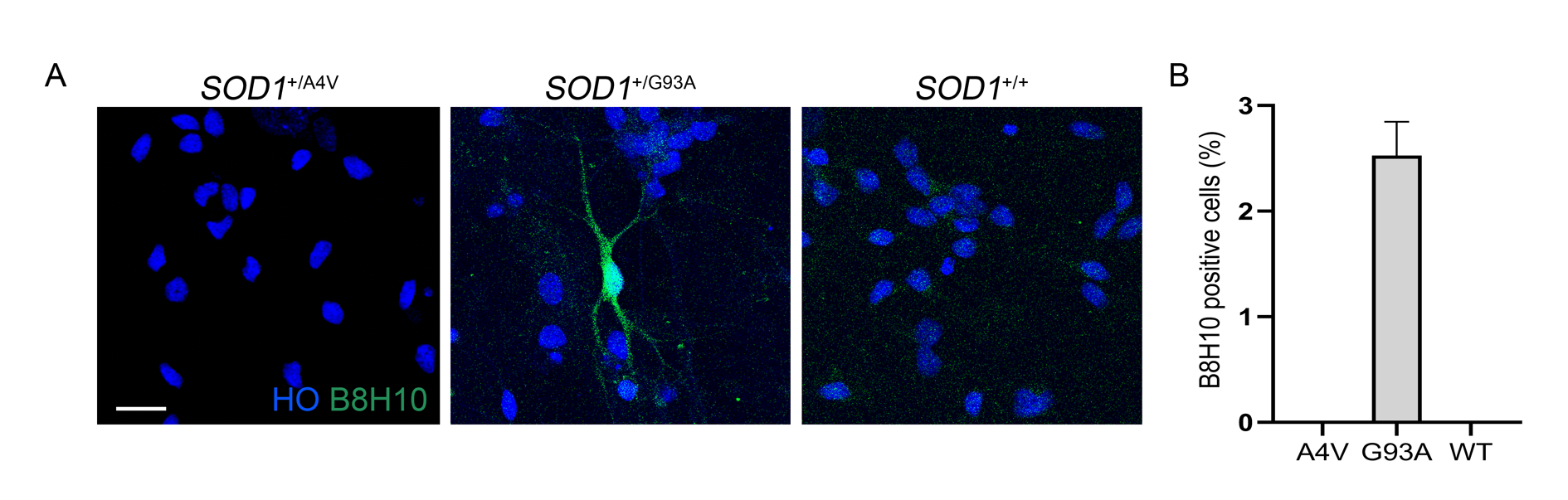

Supplement: Supplementary Figure 2 — SOD1 misfolding and aggregation detected by B8H10 antibody in MN cultures. (A) Immunofluorescence images of misfolded SOD1 detected by B8H10 monoclonal antibody in MN culture. Scale bar, 20 μm. (B) Quantification of B8H10-positive cells. n = 3 biological replicates (a total of 386 cells with SOD1+/A4V mutation, 466 cells with SOD1+/G93A mutation, and 401 cells with SOD1+/+ were evaluated). Data shown as mean ± SEM. [file Image_2.TIF]

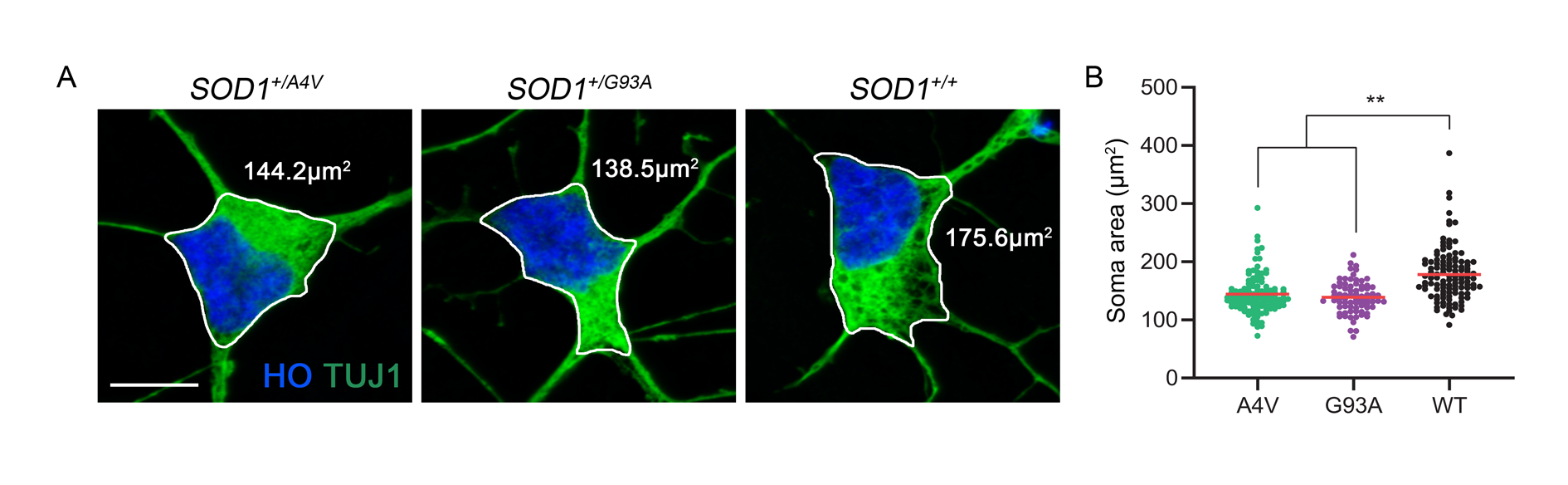

Supplement: Supplementary Figure 3 — Cell body attrition in SOD1+/A4V and SOD1+/G93A MNs. (A) Immunofluorescence images of TUJ1+ MNs with Hoechst (HO) DNA nuclear staining showing different soma sizes with contoured perimeters in white. Scale bar, 10 μm. (B) Quantification of soma size on day 28. n = 4 biological replicates (a total of 315 cells with SOD1+/A4V mutation, 206 cells with SOD1+/G93A mutation, and 266 cells with SOD1+/+ were evaluated). All data shown as mean ± SEM. ∗∗p < 0.01. [file Image_3.TIF]

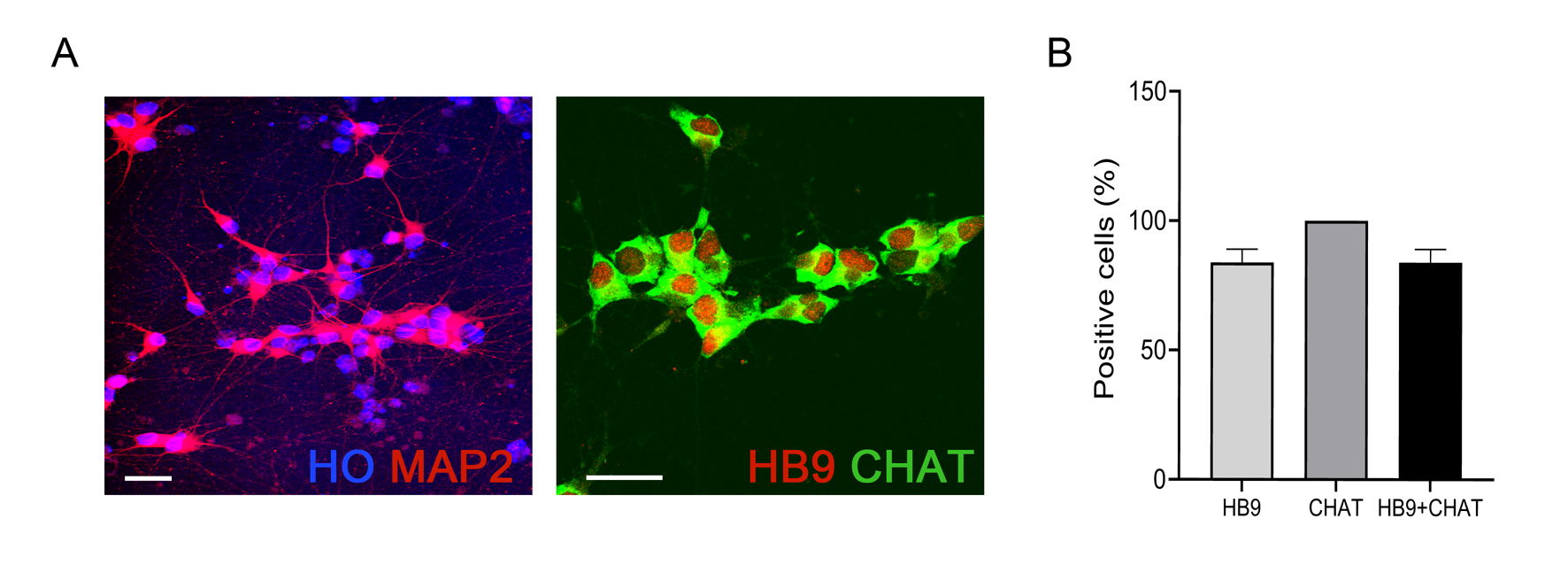

Supplement: Supplementary Figure 4 — Characterization of MNs for MEA. (A) Immuno- fluorescence images of MNs stained for MAP2, Hb9, and ChAT. Hoechst (HO) was used to counterstain the cell nuclei. Scale bar, 20 μm. (B) Quantification of MNs that are Hb9+, ChAT+, or both. All data shown as mean ± SEM. [file Image_4.TIF]

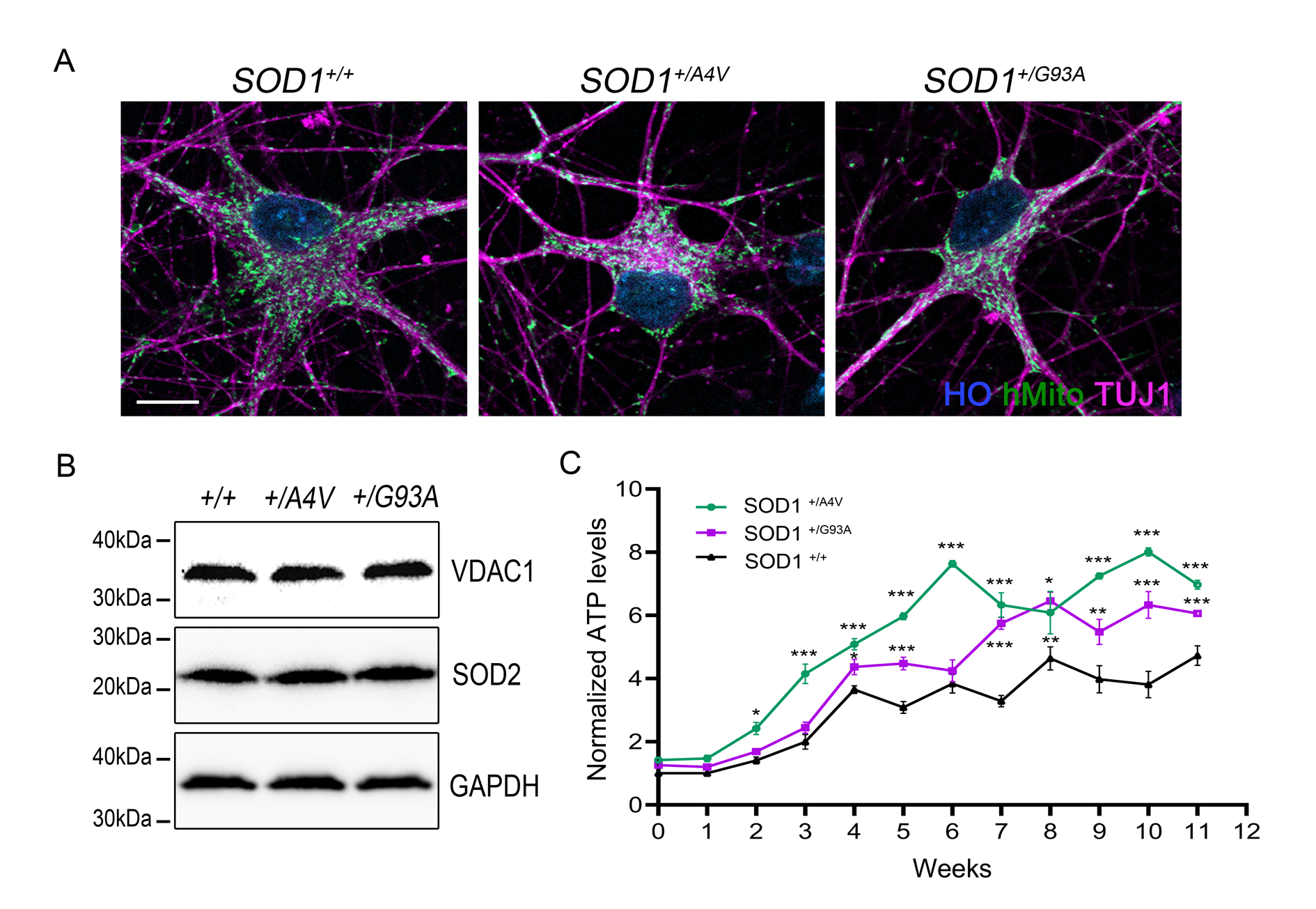

Supplement: Supplementary Figure 5 — Mitochondrial localization and ATP level in SOD1+/A4V and SOD1+/G93A MNs. (A) Immunofluorescence images of hMito+ MNs showing distribution and morphology of mitochondria. Scale bar, 10 μm. (B) Western blots showing the levels of VDAC1 and SOD2, mitochondrial markers, in whole cell lysates of MNs. GAPDH was used as a loading control. (C) Normalized ATP levels of MNs measured for 12 weeks after motor neurons became Hb9 positive. n = 8 biological replicates. All data shown as mean ± SEM. ∗p < 0.05, ∗∗p < 0.01, ∗∗∗p < 0.001. [file Image_5.TIF]
