# Supplementary material for: Human Motor Neurons With SOD1-G93A Mutation Generated From CRISPR/Cas9 Gene-Edited iPSCs Develop Pathological Features of Amyotrophic Lateral Sclerosis
Source: Front Cell Neurosci. 2020 Nov 19;14:604171. doi: 10.3389/fncel.2020.604171 (PMC7710664; doi:10.3389/fncel.2020.604171)
Supplement: Supplementary Table 1 — Human iPSC lines used to generate MNs. [file Data_Sheet_1.PDF]

Table S1. Human Induced Pluripotent Stem Cell Lines Used to Generate MNs

Gene	Mutation	iPSC lines (Clone)*	Gender	Age	Characterization*
Control	N/A	C3-1	F	40	Yes
SOD1	G93A	C3-1	F	40	N/A
SOD1	A4V	GO013	F	63	Yes

* iPSC lines, C3-1 and GO013, were provided by the Hongjun Song lab (University of Pennsylvania) and the Jeffrey Rothstein lab (Johns Hopkins University), respectively.

* Characterizations of iPSC lines, C3-1 and GO013, were previously done by the providers and include expression of pluripotency-associated markers, karyotyping, teratoma formation, genomic stability test.
