# Supplementary material for: Human Motor Neurons With SOD1-G93A Mutation Generated From CRISPR/Cas9 Gene-Edited iPSCs Develop Pathological Features of Amyotrophic Lateral Sclerosis
Source: Front Cell Neurosci. 2020 Nov 19;14:604171. doi: 10.3389/fncel.2020.604171 (PMC7710664; doi:10.3389/fncel.2020.604171)
Supplement: Supplementary Table 2 — Target and wild-type primers/probe list. [file Data_Sheet_2.PDF]

Table S2. Target and wild-type primers/probe list

ID	Sequence (5’ to 3’)	Fluorophore
SOD1-WT-probe	GATGGTGTGGC**C**GATG	HEX
SOD1-G93A-probe	GATGCTGTGGC**A**GATGT	FAM
SOD1-fwd primer	TAGGCATGTTGGAGACTT	N/A
SOD1-rev primer	CTGAGAGTGAGATCACAGA	N/A

* Gene editing site is in red and altered PAM sequence with no amino acid sequence change is in bold.
