# Supplementary material for: Human Motor Neurons With SOD1-G93A Mutation Generated From CRISPR/Cas9 Gene-Edited iPSCs Develop Pathological Features of Amyotrophic Lateral Sclerosis
Source: Front Cell Neurosci. 2020 Nov 19;14:604171. doi: 10.3389/fncel.2020.604171 (PMC7710664; doi:10.3389/fncel.2020.604171)
Supplement: Supplementary Table 3 — Primary antibody list. [file Data_Sheet_3.PDF]

Supplementary Table S3. Primary antibody list

Antibody	Host	Clone or Cat. #	Dilution	Vendor
ISL1	Mouse	39.4D5	1:100 (ICC)	DSHB
HB9	Mouse	81.5C10	1:50 (ICC)	DSHB
ChAT	Goat	AB144P	1:100 (ICC)	Millipore
MAP2	Chicken	NB300-213	1:5000 (ICC)	Novus Biologicals
MAP2	Mouse	MAB3418	1:500 (ICC)	Millipore
MAP2	Rabbit	AB5622	1:800 (ICC)	Millipore
TUJ1	Mouse	G7121, 5G8	1:2000 (ICC)	Promega
TUJ1	Rabbit	PRB-435P	1:3000 (ICC)	Covance
TAU	Chicken	AB_2313563	1:500 (ICC)	Aves Labs
SYP	Mouse	14-6525-82, EP10	1:300 (ICC)	Invitrogen
PSD95	Mouse	K28/43	1:200 (ICC)	NeuroMab
SYN	Rabbit	106002	1:500 (ICC)	Synaptic Systems
SOD1	Rabbit	Ab79390	1:5000 (WB)	Abcam
Misfolded SOD1	Mouse	C4F6	1:100 (ICC)	MediMabs
Misfolded SOD1	Mouse	B8H10	1:100 (ICC)	MediMabs
GAPDH	Mouse	10-1501, M59755	1:10,000 (WB)	Fitzgerald
VDAC1	Mouse	MSA03	1:1000 (WB)	Mitosciences
SOD2	Rabbit	ADI-SOD-111	1:1000 (WB)	Enzo
Mitochondria	Mouse	NBP2-32980	1:800 (ICC)	Novus Biologicals
Myosin	Rabbit	M7523	1:100 (ICC)	Sigma

* ICC, immunocytochemistry; WB, western blot
